# Metabolic engineering of methylotrophic *Pichia pastoris* for the production of β-alanine

**DOI:** 10.1186/s40643-021-00444-9

**Published:** 2021-09-22

**Authors:** Liangtian Miao, Yin Li, Taicheng Zhu

**Affiliations:** 1 CAS Key Laboratory of Microbial Physiological and Metabolic Engineering, State Key Laboratory of Microbial Resources, Institute of Microbiology, Chinese Academy of Sciences, Beijing, 100101 People’s Republic of China; 2 University of Chinese Academy of Sciences, Beijing, 100049 People’s Republic of China

**Keywords:** *Pichia pastoris* (*Komagataella phaffii*), β-Alanine (3-aminopropionic acid), Methanol, Aspartate decarboxylation, Aspartate dehydrogenase

## Abstract

β-Alanine (3-aminopropionic acid) is the only naturally occurring β-amino acid and an important precursor for the synthesis of a variety of nitrogen-containing chemicals. Fermentative production of β-alanine from renewable feedstocks such as glucose has attracted significant interest in recent years. Methanol has become an emerging and promising renewable feedstock for biomanufacturing as an alternative to glucose. In this work, we demonstrated the feasibility of β-alanine production from methanol using *Pichia pastoris* (*Komagataella phaffii*) as a methylotrophic cell factory. L-Aspartate-α-decarboxylases (ADCs) from different sources were screened and expressed in *P. pastoris*, followed by the optimization of aspartate decarboxylation by increasing the ADC copy number and C4 precursor supply via the overexpression of aspartate dehydrogenase. The production potential of the best strain was further evaluated in a 1-L fermenter, and a β-alanine titer of 5.6 g/L was obtained. To our best knowledge, this is the highest metabolite production titer ever reached in *P. pastoris* using methanol as the substrate.

**Graphic abstract:**

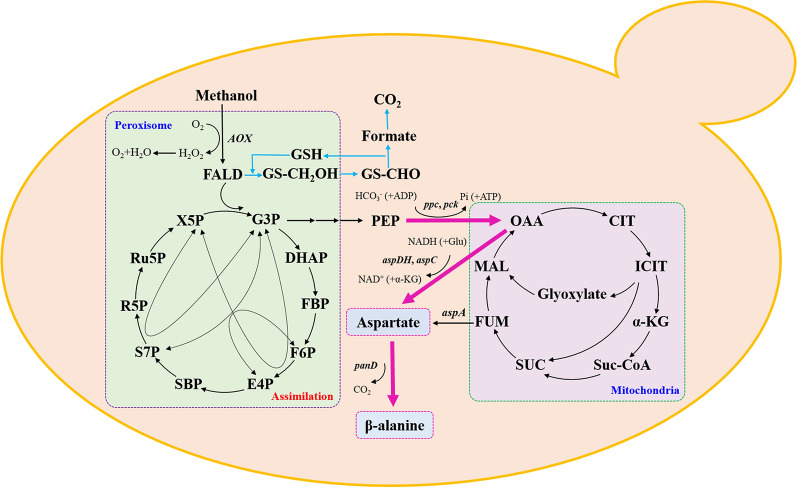

## Introduction

β-Alanine (3-aminopropionic acid) is a naturally occurring β-amino acid that serves as a precursor for the biosynthesis of a variety of nitrogen-containing chemicals, such as D-pantothenic acid (vitamin B5) (Tigu et al. 2018), coenzyme A (CoA) (Tomita et al. 2014), carnosine (Harris et al. 2006; Sale et al. 2010), and poly-alanine (nylon-3) (Steunenberg et al. 2013). β-Alanine can be chemically synthesized via the ammonification of acrylonitrile (Carlson 1943), hydrolyzation of β-aminopropionitrile in the presence of barium hydroxide (Ford 1945), or the reaction of acrylic acid with ammonium carbonate and CO_2_ (Ohara et al. 2011). However, these chemical synthesis routes are not sustainable due to the use of non-renewable substrates and harsh reaction conditions, and biological processes for β-alanine synthesis have gained increasing interest.

Biological production of β-alanine is achieved through biotransformation or fermentation. In the biotransformation route, β-alanine can be directly synthesized from l-aspartic acid via decarboxylation by l-aspartate-α-decarboxylase (ADC, EC: 4.1.1.11) (Li et al. 2018; Pei et al. 2017; Shen et al. 2014), or indirectly synthesized from fumaric acid as a cheaper substrate, via the consecutive action of aspartate ammonia-lyase (AspA, EC 4.3.1.1) and ADC (Qian et al. 2018; Wang et al. 2020). Although a very high β-alanine titer (up to 200 g/L) has been reached with whole-cell transformation, the process is not entirely sustainable due to the use of expensive precursors or petrochemicals (i.e., l-aspartic acid or fumaric acid) as substrates. Consequently, the fermentation route has been pursued to synthesize β-alanine from renewable feedstocks. The fermentative production of β-alanine from glucose has been described in several reports on the metabolic engineering of *Escherichia coli*, and promising β-alanine titers ranging from 32.3 to 43.12 g/L were obtained in these studies (Piao et al. 2019; Song et al. 2015; Zou et al. 2020).

Although glucose is the most widely used raw material for the production of biochemicals, the exploration of alternative feedstocks has remained a central task in the research on biomanufacturing (Liu et al. 2021). Methanol is considered as one of the most promising feedstocks due to its unique advantages such as providing highly reduced carbon, not competing with food sources, and potentially sustainable production in the future (Zhu et al. 2020). Consequently, there is an emerging trend of utilizing methanol as an alternative feedstock for chemical production using natural or synthetic methylotrophs (Guo et al. 2021; Jin et al. 2021; Tuyishime et al. 2018; Whitaker et al. 2017; Zhu et al. 2016).

**Fig. 1:**
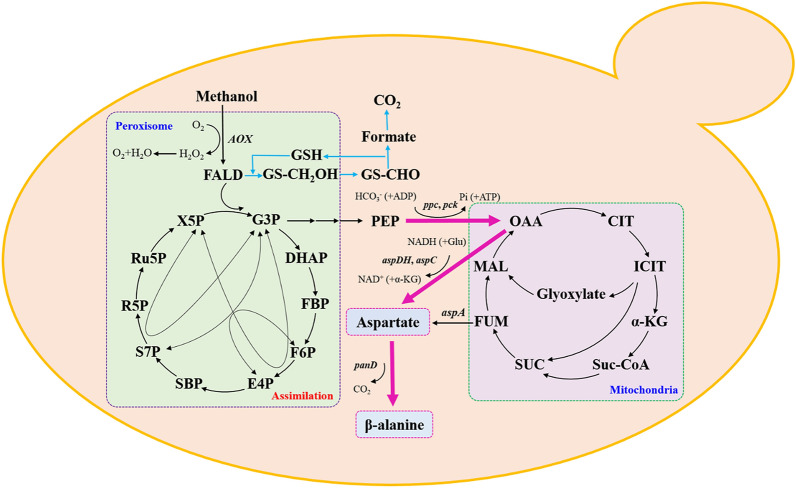
The biosynthesis pathway designed in *P. pastoris* for the production of β-alanine from methanol. *AOX*: gene encoding alcohol oxidase; *ppc*: gene encoding PEP carboxylase; *pck*: gene encoding PEP carboxykinase; *aspDH*: gene encoding aspartate dehydrogenase; *aspC*: gene encoding aspartate aminotransferase; *aspA*: gene encoding aspartate ammonia-lyase; *panD*: gene encoding aspartate-α-decarboxylases; FALD: formaldehyde; X5P: xylulose-5-phosphate; G3P: glyceraldehyde-3-phosphate; DHAP: dihydroxyacetone phosphate; FBP: fructose-1,6-bisphosphate; F6P: fructose-6-phosphate; E4P: erythrose-4-phosphate; SBP: sedoheptulose-1,7-bisphosphate; S7P: sedoheptulose-7-phosphate; R5P: ribose-5-phosphate; Ru5P: ribulose-5-phosphate; PEP: phosphoenolpyruvate; Glu: glutamate; OAA: oxaloacetate; CIT: Citrate; ICIT: isocitrate; α-KG: α-ketoglutarate; Suc-CoA: succinyl-CoA; SUC: succinate; FUM: fumarate; MAL: malate

In this work, we aimed to achieve the production of β-alanine from methanol using *Pichia pastoris* (*Komagataella phaffii*) as the methylotrophic cell chassis (Fig. 1). ADCs from different sources were screened and expressed in *P. pastoris*, followed by the optimization of aspartate decarboxylation and C4 precursor supply, and the best strain reached a β-alanine titer of 5.6 g/L. To our best knowledge, this is the first report demonstrating the feasibility of using *P. pastoris* as the chassis for the production of amino acids from methanol.

## Materials and methods

### Strains, media, and growth conditions

The primers, strains and plasmids used in this study are listed in Table 1 and Table 2. For *E*. *coli* strain construction, the cells were cultured aerobically at 37 °C in Luria–Bertani (LB) broth (10 g/L tryptone, 5 g/L yeast extract and 10 g/L NaCl). For the construction of *P. pastoris* strains, YPD (20 g/L peptone, 10 g/L yeast extract and 20 g/L glucose) or MD (20 g/L glucose, 13.4 g/L yeast nitrogen base and 0.4 mg/L biotin) medium was used, and the cells were cultured aerobically at 30 °C. Kanamycin (50 mg/L), G418 (200 mg/L), Zeocin (50 mg/L) or hygromycin (50 mg/L) were added where appropriate.

**Table 1:** Primers used in this study

Primers name	Sequences (5′ → 3′)
5′AOX1	GACTGGTTCCAATTGACAAGC
3′AOX1	GCAAATGGCATTCTGACATCC
His4-BamHI-F	GCATGGATCCATGACATTTCCCTTGCTAC
His4-BamHI-R	GCATGGATCCGTTAAATAAGTCCCAGTTTCTC
pMPICZA-3881-F	GGTTTAGTTCCTCACCTTG
pMPICZA-4256-R	TCCCCCTTTTCCTTTGTC
Kan-pMPICZA-F	ACAAGGTGAGGAACTAAACCATGAGCCATATTCAACGG
Kan-pMPICZA-R	TCGACAAAGGAAAAGGGGGATTAGAAAAACTCATCGAGC
Hyg-pMPICZA-F	ACAAGGTGAGGAACTAAACCATGAAAAAGCCTGAACTCAC
Hyg-pMPICZA-R	TCGACAAAGGAAAAGGGGGACTATTCCTTTGCCCTCGG
pTEF1-F	GCAATCTAATCTAAGGGGCGGTGT
CYC1TT-R	TTCGAGCGTCCCAAAACCTTCTCAAGC
ADCBs-XhoI-F	GCATCTCGAGATGGATGTATCGAACAATGATGAGC
ADCBs-NotI-R	GCATGCGGCCGCCTACAAAATTGTACGGGCTG
Oan-XhoI-F	GCATCTCGAGATGTCCGTATCTGAAACC
Oan-NotI-R	GCATGCGGCCGCTCAGATAACGGTAGTTGCTACGC
Spe-XhoI-F	GCATCTCGAGATGAAAAAAATCATGATGATCGG
Spe-NotI-R	GCATGCGGCCGCTTATGCGATAAAGCCACCGTC
AAT1-BstBI(gb)-F	ATCAAAAAACAACTAATTATTCGAAATGTCGTTTTCAACGCAGAA
AAT1-NotI(gb)-R	AGTTTTTGTTCTAGAAAGCTGGCGGCCGCCTATACACGCACAACCTGAT
AAT2-BstBI(gb)-F	ATCAAAAAACAACTAATTATTCGAAATGTCGTTTTCAACGCAGAA
AAT2-NotI(gb)-R	AGTTTTTGTTCTAGAAAGCTGGCGGCCGCCTATACACGCACAACCTGAT
AsPCK-BstBI(gb)-F	ATCAAAAAACAACTAATTATTCGAAATGACTGACTTAAACAAACTCG
AsPCK-NotI(gb)-R	AGTTTTTGTTCTAGAAAGCTGGCGGCCGCTTATGCTTTTGGACCGGCGCCAAC
PPC-BstBI(gb)-F	ATCAAAAAACAACTAATTATTCGAAATGAACGAACAATATTCCGC
PPC-NotI(gb)-R	AGTTTTTGTTCTAGAAAGCTGGCGGCCGCTTAGCCGGTATTACGCATAC
PCK-BstBI(gb)-F	ATCAAAAAACAACTAATTATTCGAAATGCGCGTTAACAATGGTTTG
PCK-NotI(gb)-R	AGTTTTTGTTCTAGAAAGCTGGCGGCCGCTTACAGTTTCGGACCAGCCGC

**Table 2:** Plasmids and strains constructed in this study

Names	Relative characteristics	References
*Plasmids*		
pPICZA	Vector for extracellular expression recombinant protein carrying Zeo^R^	Invitrogen
pAOα	Vector for extracellular expression; derived from pPICZαA and pAO815, *HIS4* +	Zhu et al. (2011)
pMPICZHis	Extracellular expression vector carrying *HIS4* gene, Zeo^R^	This work
pMPICKmHis	Replace antibiotic resistance marker (Zeocin) of pMPICZHis with kanamycin	This work
pMPIC2H	Replace antibiotic resistance marker (Zeocin) of pMPICZHis with hygromycin	This work
pMPICZHis-bliADC	Codon-optimized *panD* gene from *Bacillus paralicheniformis* ATCC 9945a cloned into pMPICZHis at *Bst*BI and *Not*I site	This work
pMPICZHis-bsADC	Codon-optimized *panD* gene from *Bacillus subtilis* cloned into pMPICZHis at *Bst*BI and *Not*I site	This work
pMPICZHis-sgeADC	Codon-optimized *panD* gene from *Streptomyces griseorubiginosus* cloned into pMPICZHis at *Bst*BI and *Not*I site	This work
pMPICZHis-cguADC	Codon-optimized *panD* gene from *Corynebacterium glutamicum* cloned into pMPICZHis at *Bst*BI and *Not*I site	This work
pMPICZHis-TcCSADC	Cysteine sulfinic acid decarboxylase from *Tribolium castaneum* (TcCSADC) cloned into pMPICZHis at *Bst*BI and *Not*I site	This work
pMPICZHis-Oan	*aspDH* gene from *Ochrobactrum anthropi* ATCC 4918 cloned into pMPICZHis at *Bst*BI and *Not*I site	This work
pMPICZHis-Spe	*aspDH* gene from *Serratia proteamaculans* cloned into pMPICZHis at *Bst*BI and *Not*I site	This work
pMPICZHis-ADC-Oan	*panD* expression cassette (pMPICZHis-ADC digest by *Sph*I and *Bam*HI) cloned into pMPICZHis-Oan at *Sph*I and *Bgl*II site	This work
pMPICZHis-ADC-Spe	*panD* expression cassette (pMPICZHis-ADC digest by *Sph*I and *Bam*HI) cloned into pMPICZHis-Spe at *Sph*I and *Bgl*II site	This work
pMPICKmHis-AAT1	Cytosolic aspartate aminotransferase from *P*. *pastoris* GS115 cloned into pMPICZHis at *Bst*BI and *Not*I site	This work
pMPICKmHis-AAT2	Mitochondrial aspartate aminotransferase from *P*. *pastoris* GS115 cloned into pMPICZHis at *Bst*BI and *Not*I site	This work
pMPICZHis-2ADC	Two copies of *panD* gene expression cassette cloned into pMPICZHis	This work
pMPICZHis-2ADC-Spe	Two copies of *panD* gene expression cassette and one copy of *SpeaspDH* gene expression cassette cloned into pMPICZHis	This work
pMPIC2H-PPC	*ppc* gene from *Escherichia coli* cloned into integration vector pMPIC2H at *Bst*BI and *Not*I site	This work
pMPIC2H-AsPCK	*pck* gene from *Actinobacillus succinogenes* cloned into integration vector pMPIC2H at *Bst*BI and *Not*I site	This work
pMPIC2H-PCK	*pck* gene from *Escherichia coli* cloned into integration vector pMPIC2H at *Bst*BI and *Not*I site	This work
*Strains*		
*E*. *coli* DH5α	Commercial transformation host for cloning	Invitrogen
*P*. *pastoris* GS115	Commercial transformation host for cloning; *HIS4*-, Mut +	Invitrogen
Δku70	GS115, Δ*KU70*; *HIS4* +	This work
BliADC	Δku70 harboring pMPICZHis-bliADC	This work
BsADC	Δku70 harboring pMPICZHis-bsADC	This work
SgeADC	Δku70 harboring pMPICZHis-sgeADC	This work
CguADC	Δku70 harboring pMPICZHis-cguADC	This work
TcCSADC	Δku70 harboring pMPICZHis-TcCSADC	This work
ADC-Oan	Δku70 harboring pMPICZHis-ADC-Oan	This work
ADC-Spe	Δku70 harboring pMPICZHis-ADC-Spe	This work
ADC-AAT1	Δku70 harboring pMPICZHis-bsADC and pMPICKmHis-AAT1	This work
ADC-AAT2	Δku70 harboring pMPICZHis-bsADC and pMPICKmHis-AAT2	This work
2ADC	Δku70 harboring pMPICZHis-2ADC	This work
2ADC-Spe	Δku70 harboring pMPICZHis-2ADC-Spe	This work
PPC	Δku70 harboring pMPICZHis-2ADC-Spe and pMPIC2H-PPC	This work
AsPCk	Δku70 harboring pMPICZHis-2ADC-Spe and pMPIC2H-AsPCK	This work
PCK	Δku70 harboring pMPICZHis-2ADC-Spe and pMPIC2H-PCK	This work

### Construction of recombinant plasmids

The *P. pastoris HIS4* gene was amplified by PCR from pAOα vector (Zhu et al. 2011) with the primer pair His4-BamHI-F/His4-BamHI-R, which was then digested with *Bam*HI and ligated into the *Bam*HI site of pPICZA to generate the expression vector pMPICZHis. The zeocin resistance marker of pMPICZHis was replaced with the kanamycin or hygromycin resistance gene using a Gibson assembly strategy to yield the expression vectors pMPICKmHis and pMPIC2H, respectively. The *panD* genes encoding ADCs from *Bacillus paralicheniformis* ATCC 9945a (GenBank: AGN36811.1), *Bacillus subtilis* (NCBI Reference Sequence: WP_161619406.1), *Streptomyces griseorubiginosus* (NCBI Reference Sequence: WP_120050174.1), and *Corynebacterium glutamicum* (NCBI Reference Sequence: WP_003857183.1), the *aspDH* genes encoding aspartate dehydrogenase (AspDH) from *Ochrobactrum anthropi* ATCC 4918 (GenBank: ABS17096.1) and *Serratia proteamaculans* (NCBI Reference Sequence: WP_174354643.1), as well as the gene encoding cysteine sulfinic acid decarboxylase from *Tribolium castaneum* (NCBI Reference Sequence: XM_966741.3) were codon optimized, synthesized and subcloned into pMPICZHis between the *Bst*BI and *Not*I sites. The genes encoding cytosolic and mitochondrial aspartate aminotransferases (AAT) were PCR amplified from genomic DNA of *P. pastoris* GS115 using primers pair AAT1-BstBI(gb)-F/AAT1-NotI(gb)-R and AAT2-BstBI(gb)-F/AAT2-NotI(gb)-R, which was then cloned into pMPICKmHis between the *Bst*BI and *Not*I sites using Gibson assembly method. Similarly, the *ppc* and *pck* genes from *E*. *coli* BW25113 (GenBank: CP072663.1), as well as the *pck* gene from *Actinobacillus succinogenes* (GenBank: AY308832.1) were PCR amplified via the primers pair PPC-BstBI(gb)-F/PPC-NotI(gb)-R, PCK-BstBI(gb)-F/PCK-NotI(gb)-R and AsPCK-BstBI(gb)-F/AsPCK-NotI(gb)-R and cloned into pMPIC2H between the *Bst*BI and *Not*I sites.

A previously described multi-copy plasmid construction method (Yu et al. 2020a) was applied for the construction of the 2-, or 3-gene co-expression plasmids. For example, the pMPICZHis-ADC plasmid was double-digested with *Sph*I and *Bam*HI to generate the expression cassette 5’AOX1-ADC-3’AOX1, which was then inserted between the *Sph*I and *Bgl*II sites of pMPICZHis-ADC to create pMPICZHis-2ADC. Similarly, the gene cassette 5’AOX1-Spe-3’AOX1 was reinserted between the *Sph*I and *Bgl*II sites of pMPICZHis-2ADC to create pMPICZHis-2ADC-Spe.

### Construction of recombinant strains

The *KU70* gene of the wild-type *P. pastoris* strain GS115 was deleted using the Cre-LoxP method to improve the homologous recombination efficiency (Guo et al. 2021; Weninger et al. 2018). The expression vectors pMPICZHis-bliADC, pMPICZHis-bsADC, pMPICZHis-sgeADC, pMPICZHis-cguADC, pMPICZHis-TcCSADC, pMPICZHis-ADC-Oan, pMPICZHis-ADC-Spe, pMPICZHis-2ADC, and pMPICZHis-2ADC-Spe were linearized with *Bsp*EI and used to transform the Δku70 strain by electroporation. The linearized vectors were integrated into the chromosome of Δku70 strain at the *HIS4* gene locus. After integration of the linearized vector, the mutant *HIS4* gene was repaired, enabling the recombinant strains growing on MD plate. Then, 8–16 colonies were picked for PCR verification and 3 correct colonies were randomly selected for fermentation analysis. Positive transformants were named BliADC, BsADC, SgeADC, CguADC, TcCSADC, ADC-Oan, ADC-Spe, 2ADC and 2ADC-Spe, respectively. The expression vectors pMPICKmHis-AAT1 and pMPICKmHis-AAT2 were linearized using *Bsp*EI and used to transform the BsADC strain to generate ADC-AAT1 and ADC-AAT2, respectively. The expression vectors pMPIC2H-PPC, pMPIC2H-AsPCK, and pMPIC2H-PCK were linearized with *Bsp*EI, *Stu*I and *Ssp*I, respectively, and used to transform the strain 2ADC-Spe, yielding the recombinant strains PPC, AsPCK and PCK, respectively.

### β-Alanine production in shake-flask fermentations

The strains were precultured in 10 mL YPD medium for 48 h. This seed culture was centrifuged and resuspended with 25 mL of BMMY medium (20 g/L peptone, 10 g/L yeast extract, 13.4 g/L YNB, 0.4 mg/L biotin, 8.7 g/L monobasic potassium phosphate, pH 6.0) in 250-mL baffled shake flasks with an initial OD_600_≈3.0. The fermentation was carried out at 30 °C and 220 rpm for 6 days. Recombinant gene expression was induced by adding 200 μL of pure methanol to each flask, followed by feeding with 200 µL of methanol at 12 h intervals.

### β-Alanine production by fed-batch fermentation in a 1-L fermenter

The strains were first grown in 50 mL YPD for 36 h and transferred into 1-L stirred tank reactors (Infors, Switzerland) containing 0.8 L of BMGY (20 g/L peptone, 10 g/L yeast extract, 13.4 g/L YNB, 0.4 mg/L biotin, 8.7 g/L monobasic potassium phosphate, 40 g/L glycerol, pH 6.0) supplemented with 4.0 mL PTM1 trace salts (6 g/L CuSO_4_·5H_2_O, 0.09 g/L KI, 3 g/L MnSO_4_·H_2_O, 0.02 g/L H_3_BO_3_, 0.2 g/L MoNa_2_O_4_·2H_2_O, 0.5 g/L CoCl_2_, 20 g/L ZnCl_2_, 65 g/L FeSO_4_·7H_2_O, 0.2 g/L biotin, 5.0 mL/L H_2_SO_4_). The temperature was set to 30 °C, the pH was controlled at 6.0 by adding NH_3_⋅H_2_O (28%, v/v), the dissolved oxygen concentration was kept above 20% of the atmospheric value by varying the air flow rate between 0.5 and 2 L/min. The entire cultivation started with a batch phase lasting for 20 − 24 h. Heterologous gene expression was induced by the addition of 0.25% (v/v) methanol, and the methanol concentration was maintained at 3 g/L throughout the entire fermentation span using an automatic methanol control station (FC2002, East China University of Science and Technology, Shanghai, China).

### Analytical methods

The cell growth was analyzed by measuring the optical density at 600 nm. β-Alanine production was measured by high-performance liquid chromatography (HPLC) with a variable wavelength detector (VWD) set to 334 nm and an Agilent ZRABOX SB-C18 column (4.6 mm × 250 mm, 5 μm) after centrifugation of the fermentation samples and o-phthalaldehyde (OPA) derivatization (Pei et al. 2017). The mobile phase consisted of 35 mM sodium acetate (pH 7.5) containing 30% methanol with a flow rate of 1 mL/min.

## Results

### Overexpression of ADCs from different sources for the production of β-alanine and tolerance of the *P. pastoris* chassis to β-alanine

**Fig. 2:**
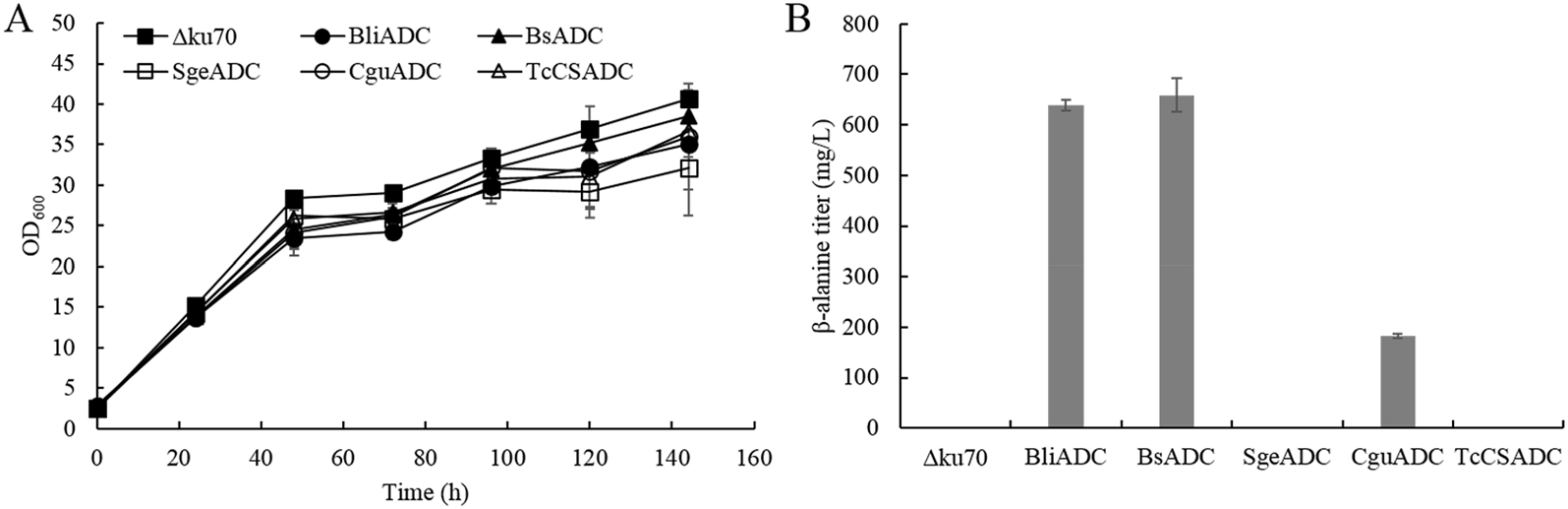
Overexpression of ADCs from different sources for β-alanine synthesis. **A** Growth profiles of recombinant strains; **B** β-alanine production in recombinant strains at the end of fermentation. Three parallel flasks are tested for each strain. Error bars represent deviations (*n* = 3)

**Fig. 3:**
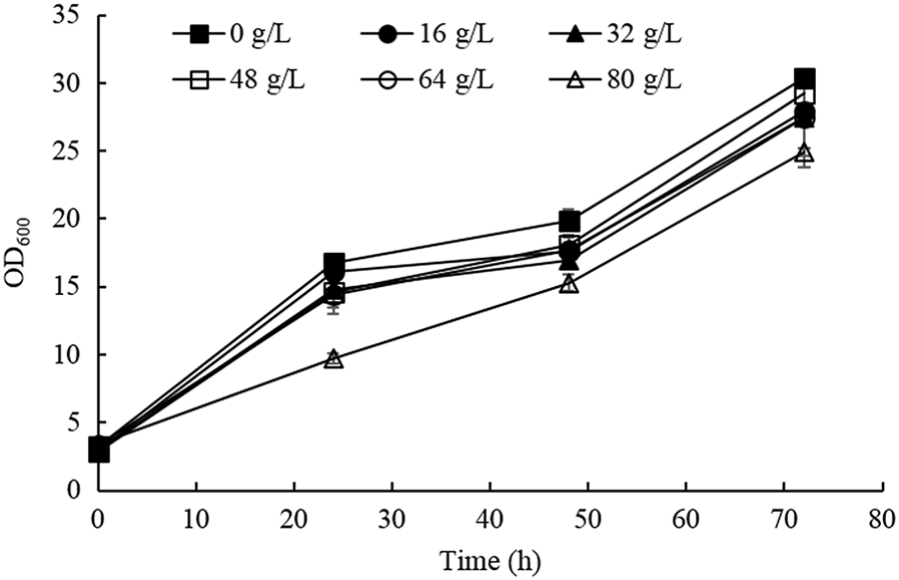
Tolerance of wild-type *P. pastoris* strain for various concentrations of β-alanine

Since aspartate is the direct precursor for β-alanine synthesis via decarboxylation by ADCs, and was reported to have one of the largest precursor pool sizes among all amino acids in *P. pastoris* (Carnicer et al. 2012), we hypothesized that overexpression of ADC would lead to the accumulation of β-alanine. Nevertheless, only trace amount of extracellular aspartate (~ 80 mg/L) were detected for the wild-type *P. pastoris* (data not shown) probably because aspartate is an intermediate metabolite and proteinogenic amino acid and thus is not prone to accumulate. Four genes encoding ADC from *B. paralicheniformis*, *B. subtilis*, *S. griseorubiginosus*, *C. glutamicum*, as well as the gene encoding cysteine sulfinic acid decarboxylase from *T*. *castaneum*, which was reported to possess higher decarboxylation activity than ADCs, were evaluated in this work. The coding sequences were individually placed under the control of the strong *AOX1* promoters and integrated into the genome of *P. pastoris*. In order to increase the efficiency of homologous recombination (HR), the *KU70* mutant strain (Δku70) which has impaired nonhomologous end joining (NHEJ) was used as a parent strain (Guo et al. 2021; Weninger et al. 2018). The individual overexpression of ADCs from *B*. *paralicheniformis*, *B*. *subtilis* and *C*. *glutamicum* enabled the accumulation of β-alanine after 6 days of fermentation when feeding the strains with methanol (Fig. 2B), indicating the functional protein expression of ADCs. The highest titer (658.9 mg/L) was reached by the recombinant strain expressing *B*. *subtilis* ADC (BsADC) (Fig. 2B), and BsADC was used as the starting strain for further metabolic engineering. To investigate whether the tolerance to β-alanine was a limiting factor for β-alanine production, *P. pastoris* was cultivated in media supplemented with different concentrations of β-alanine. The results showed that yeast cell growth was substantially reduced only in the presence of 80 g/L of β-alanine (*P* < 0.01) at 72 h, while the cell growth at other concentrations was not significantly different from the control (*P* > 0.05) (Fig. 3).

### Overexpression of AAT or AspDH to increase aspartate supply for improved β-alanine synthesis

**Fig. 4:**
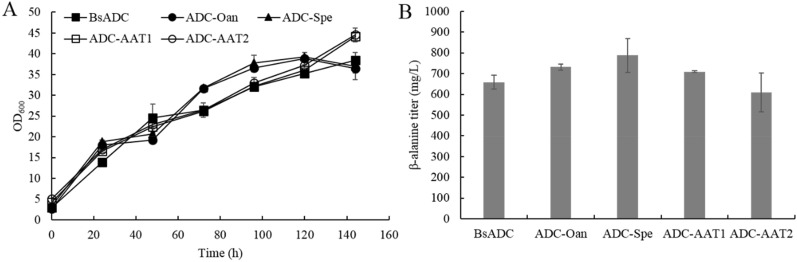
The effects of overexpression of AAT or AspDH on β-alanine production. **A** Growth profiles of recombinant strains; **B** β-alanine production in recombinant strains at the end of fermentation. Three parallel flasks are tested for each strain. Error bars represent deviations (*n* = 3)

The conversion of oxaloacetate (OAA) into aspartate can be achieved by the transfer of an amino group from glutamate catalyzed by AAT, or direct amination with ammonium as the amino group donor catalyzed by AspDH. *P. pastoris* possesses a mitochondrial AAT (encoded by *AAT1*) and a cytosolic AAT (*AAT2*), and both genes were cloned into the pMPICKmHis vector and tested for their effects on β-alanine synthesis, respectively. In a previous paper, we reported that an AspDH from *S. proteamaculans* has high activity and stability (Li et al. 2017a). Thus, the SpeAspDH was also overexpressed in recombinant *P. pastoris* using the same strategy as AAT1 and AAT2. The shake-flask fermentation results showed that while neither AAT1 or AAT2 significantly improved β-alanine synthesis, the β-alanine titer was increased by 19.6% (to 787.9 mg/L) by overexpressing SpeAspDH (Fig. 4B). In addition, no extracellular aspartate accumulation could be detected for all ADC overexpressed strains even after the optimization of the aspartate precursor supply (data not shown).

### Further improvement of β-alanine production by increasing the ADC copy number

**Fig. 5:**
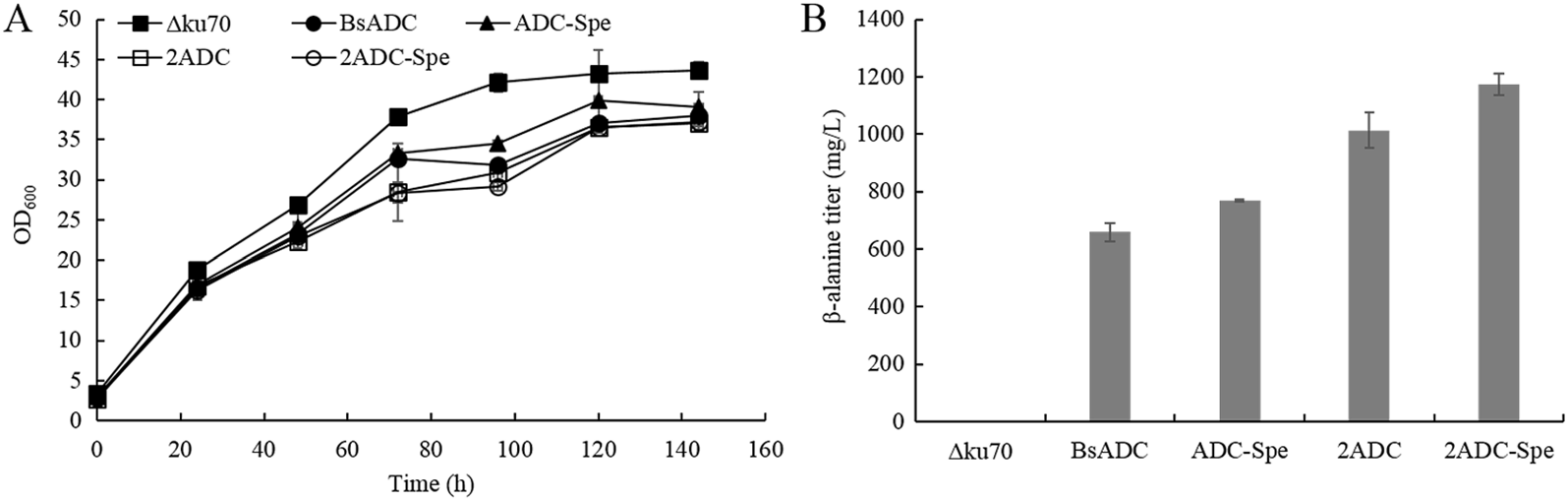
The effects of increasing ADC copy number on β-alanine production. **A** Growth profiles of recombinant strains; **B** β-alanine production in recombinant strains at the end of fermentation. Three parallel flasks are tested for each strain. Error bars represent deviations (*n* = 3)

After optimization of the aspartate precursor supply, we investigated whether aspartate decarboxylation is a potential bottleneck for β-alanine production by increasing the copy number of the encoding gene. Vectors harboring two copies of the ADC expression cassette alone and together with the SpeAspDH expression cassette were introduced into the Δku70 strain, resulting in the recombinant strains 2ADC and 2ADC-Spe, respectively. The multi-copy strains 2ADC and 2ADC-Spe exhibited 53.9 and 52.6% increases of β-alanine production compared with their respective single-copy counterparts, ADC and ADC-Spe (Fig. 5B). However, increase of ADC copy number to three did not lead to further increase of β-alanine (data not shown). Notably, the cell growth of all β-alanine producing strains (especially the two copy strains) were significantly slower than that of the wild-type strain (Fig. 5A), illustrating the negative effects of ADC overexpression on the physiology of yeast cells.

### Effect of strengthening phosphoenolpyruvate (PEP) carboxylation on β-alanine production

**Fig. 6:**
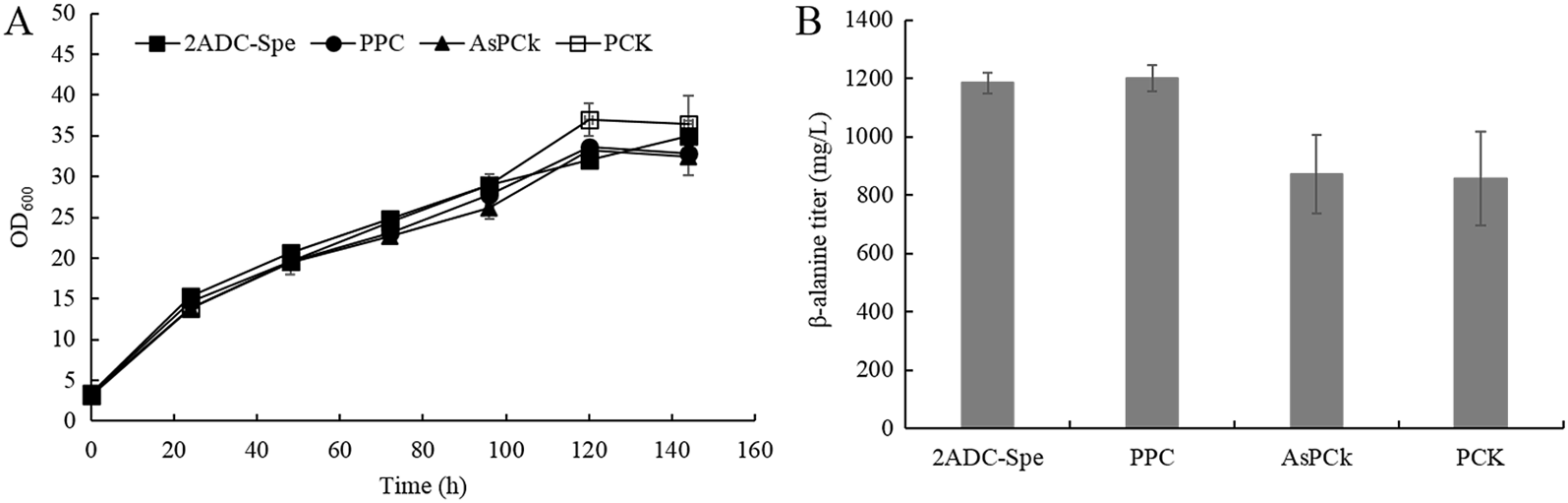
The effects of strengthening PEP carboxylation on β-alanine production. **A** Growth profiles of recombinant strains; **B** β-alanine production in recombinant strains at the end of fermentation. Three parallel flasks are tested for each strain. Error bars represent deviations (*n* = 3)

A number of studies showed that CO_2_ fixation-based carboxylation of C3 metabolites plays an important role in the synthesis of C4 precursors (Tan et al. 2013). Therefore, the effect of strengthening PEP carboxylation on C4 precursor supply and thereby β-alanine synthesis was further investigated. Since PEP can be converted into OAA by either PEP carboxylase (PPC) or PEP carboxykinase (PCK), the encoding *ppc* and *pck* genes from *E*. *coli* (Zhang et al. 2011), as well as *pck* gene (encoding the ATP forming PEP carboxykinase, E.C: 4.1.1.49) from *A*. *succinogenes* (Hu et al. 2018), were individually overexpressed in 2ADC-Spe to evaluate their impact on β-alanine production in shake-flask fermentations. However, overexpression of *ppc* did not lead to significant increase in the β-alanine titer compared to the control (2ADC-Spe), while both PCKs from *A*. *succinogenes* and *E*. *coli* decreased the β-alanine titer (by 26.4 and 27.6%, respectively) (Fig. 6B).

### Fed-batch fermentation to improve β-alanine production

**Fig. 7:**
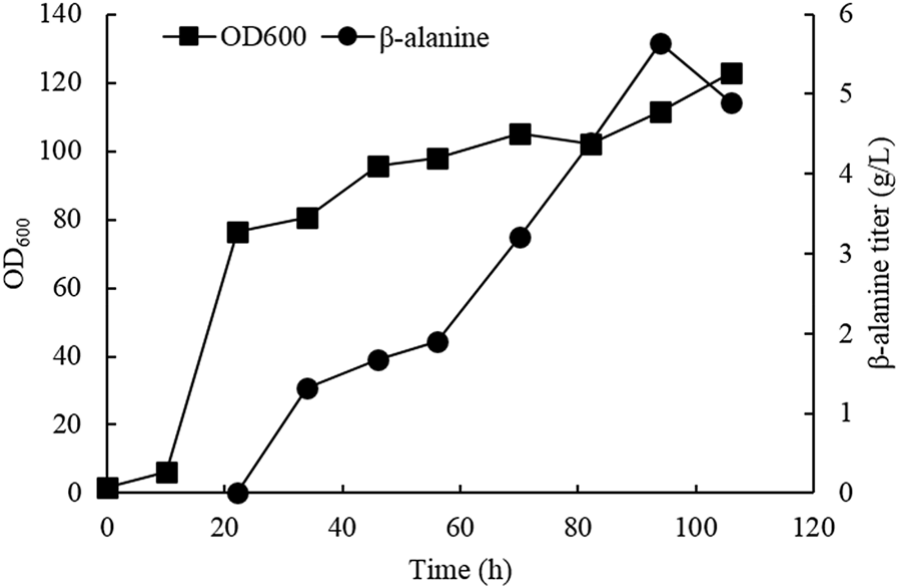
Fed-batch fermentation profile of 2ADC-Spe strain

In order to obtain a higher β-alanine titer, fed-batch fermentation of the 2ADC-Spe strain was performed in a 1-L fermenter using a two-stage strategy. The fermentation was started with a glycerol phase (40 g/L glycerol) to facilitate biomass accumulation. When the glycerol was depleted and an OD_600_ of approximately 80 was reached, the methanol phase was induced by maintaining the methanol concentration at 3 g/L using an on-line methanol analyzer. β-Alanine accumulation began in the methanol phase, and the highest β-alanine titer reached 5.6 g/L (Fig. 7). However, the yeast cells grew very slowly during the entire methanol phase and the biomass only reached an OD_600_ of 123 after 106 h (Fig. 7), corresponding to an average specific growth rate of 0.0044 h^−1^.

## Discussion

*Pichia pastoris* is one of the most widely used eukaryotic expression systems for heterologous proteins, and in recent years, its potential as a cell factory for the production of chemicals is also receiving increasing attention (Zhu et al. 2019). As a native methylotroph, *P. pastoris* has unique advantages over *S. cerevisiae* and *E. coli* when methanol is used as a feedstock (Siripong et al. 2020; Siripong et al. 2018). Nevertheless, it is still challenging to engineer *P. pastoris* for the production of chemicals, largely because as a Crabtree-negative yeast, *P. pastoris* tends to accumulate biomass rather than produce metabolites. Reports on metabolite production in fermentations of engineered *P. pastoris* mainly remain at the proof-of-concept stage, with titers usually lower than 1 g/L, especially when methanol is used as substrate (Gao et al. 2021). Several recent studies reported gram per liter metabolites production in test tubes or shake flasks. Yamada et al. (2019) reported a 3.48 g/L of d-lactic acid production from 30 g/L of methanol by engineered *P. pastoris* using YPM medium containing yeast extract and peptone. With an additional induction step by culturing engineered yeast cells in methanol complex medium (BMMY medium), Guo et al. (2021) achieved 0.75 g/L and 2.79 g/L of malic acid production in mineral-salt based fermentation medium with and without the addition of 1 g/L of yeast extract, respectively. Unfortunately, the chemicals production potential of these recombinant *P. pastoris* strains was not fully evaluated by cultivation on the fermenter scale. In this study, we achieved a β-alanine titer of 1.2 g/L using BMMY medium in shake-flask culture, which is the first demonstration of amino acids production from methanol with *P. pastoris* as the chassis. More importantly, the production of the strain was further evaluated in a 1-L fermenter using a two-stage strategy with a high initial biomass. Finally, 5.6 g/L of β-alanine titer was obtained, which represents the highest metabolite production titer ever reached in *P. pastoris* using methanol as the substrate.

Aspartate decarboxylation is the most important step of β-alanine synthesis. Although ADCs from different sources have been evaluated for β-alanine synthesis in *E. coli* (Feng et al. 2019; Liu et al. 2019; Pei et al. 2017; Song et al. 2015), the protein expression levels and enzyme activities of these ADCs may be different in eukaryotic hosts such as *P. pastoris*. Accordingly, we evaluated bacterial ADCs from different sources, including ones known to function well in *E. coli* such as the ADCs from *C. glutamicum* (Song et al. 2015) and *B. subtilis* (Pei et al. 2017), as well as the recently reported insect TcCSADC, which is reported to be a dimer that is resistant to turnover-dependent inactivation observed in ADCs from prokaryotes (Liu et al. 2019; Yu et al. 2020b). Only ADCs from *Bacillus* spp. exhibited high activity in the *P. pastoris* system, and the mere expression of *B. subtilis* ADC resulted in an initial success in β-alanine accumulation. Moreover, we showed that β-alanine production can be remarkably improved by doubling the ADC copy number, indicating that even with the strong *AOX1* promoter, the decarboxylation of aspartate still remains the bottleneck for β-alanine synthesis. Increasing the copy number of the target gene is a widely used strategy in recombinant protein expression in *P. pastoris* (Yu et al. 2020a). Our work suggested that this strategy is still important in tuning metabolic pathways for chemical synthesis.

Increasing the aspartate supply is also crucial for achieving high β-alanine production. To this end, two strategies were applied. First, we aimed at increasing the conversion of OAA into aspartate by screening for appropriate enzymes for reductive amination. Although the overexpression of AAT was widely used to increase aspartate flux for the production of aspartate family amino acids (AFAAs) in industrial microbes such as *C. glutamicum* and *E. coli* (Li et al. 2017a, b; Piao et al. 2019), the efficacy of AspDH overexpression for enhancing AFAAs production was rarely. The present study demonstrates that the overexpression of SpeAspDH can significantly increase β-alanine production, corroborating the potential of AspDHs for the production of AFAAs. The second strategy is to increase OAA supply by strengthening C3 carboxylation. PPC exhibits high affinity for bicarbonate and high catalytic velocity in the carboxylation of PEP, but the energy contained in PEP is lost in this reaction with the release of inorganic phosphate. Conversely, PCK can conserve the high energy of PEP, leading to net production of ATP for growth and cell maintenance, but it has low affinity for bicarbonate and relatively low catalytic velocity (Tan et al. 2013). In this study, the overexpression of PPC from *E*. *coli* only led to a slight increase of the β-alanine titer, and *pck* overexpression even decreased β-alanine production, suggesting that OAA supply may not be the bottleneck for β-alanine synthesis at the current stage, and there might be other rate-limiting factors that should be resolved in future studies.

Another noteworthy phenomenon observed in this work is that the growth of the cells was remarkably decreased, especially for strains harboring two copies of ADC. After shifting to the methanol phase in the fermenter, the yeast cells grew very slowly, with an average specific growth rate of 0.0044 h^−1^, which was an order of magnitude lower than that of the wild-type strain under the same conditions (usually more than 0.04 h^−1^). The impaired cell growth of the ADC-expressing strain is less likely caused by product inhibition, because the β-alanine titer far below its inhibitory concentration. Nevertheless, this phenomenon is not quite unexpected because overexpression of ADC may cause considerable metabolic burden on *P. pastoris*. Moreover, as an important metabolic intermediate, aspartate takes part in many biological processes, such as the synthesis of AFAAs (Li et al. 2017b; Park and Lee 2010), protecting microbes against acid stress (Wu et al. 2013), shuttling of redox equivalents (Bakker et al. 2001), etc., and depletion of aspartate for β-alanine synthesis may cause severe perturbations of the normal physiology of yeast cells. The underlying mechanism is currently under investigation using omics approaches.

## Conclusions

Recombinant *P. pastoris* strains were constructed for the production of β-alanine from methanol by screening and overexpressing ADCs from different sources. The β-alanine titer was further increased by increasing ADC copy number and overexpression of AspDH. The production potential of the best strain 2ADC-Spe was evaluated on 1-L fermenter and 5.6 g/L of β-alanine titer was obtained, which is the highest metabolite production titer ever reached in *P. pastoris* using methanol as the substrate. This work is the first attempt to produce amino acids from methanol using recombinant *P. pastoris* as the cell chassis.

## Data Availability

All data generated or analyzed during this study are included in this article.

## Abbreviations

ADC: Aspartate-α-decarboxylases
AspA: Aspartate ammonia-lyase
AAT: Aspartate aminotransferase
AspDH: Aspartate dehydrogenase
HR: Homologous recombination
NHEJ: Nonhomologous end joining
OAA: Oxaloacetate
PEP: Phosphoenolpyruvate
PPC: PEP carboxylase
PCK: PEP carboxykinase
AFAAs: Aspartate family amino acids
AOX: Alcohol oxidase
